# Gene Duplication of the Zebrafish *kit ligand* and Partitioning of Melanocyte Development Functions to *kit ligand a*

**DOI:** 10.1371/journal.pgen.0030017

**Published:** 2007-01-26

**Authors:** Keith A Hultman, Nathan Bahary, Leonard I Zon, Stephen L Johnson

**Affiliations:** 1 Department of Genetics, Washington University School of Medicine, Saint Louis, Missouri, United States of America; 2 Department of Molecular Genetics and Biochemistry, University of Pittsburgh School of Medicine, Pittsburgh, Pennsylvania, United States of America; 3 Department of Pediatrics, Children's Hospital, Boston, Massachusetts, United States of America; 4 Howard Hughes Medical Institute, Boston, Massachusetts, United States of America; Stanford University School of Medicine, United States of America

## Abstract

The retention of particular genes after the whole genome duplication in zebrafish has given insights into how genes may evolve through partitioning of ancestral functions. We examine the partitioning of expression patterns and functions of two zebrafish *kit ligands, kit ligand a (kitla)* and *kit ligand b (kitlb),* and discuss their possible coevolution with the duplicated zebrafish *kit* receptors *(kita* and *kitb).* In situ hybridizations show that *kitla* mRNA is expressed in the trunk adjacent to the notochord in the middle of each somite during stages of melanocyte migration and later expressed in the skin, when the receptor is required for melanocyte survival. *kitla* is also expressed in other regions complementary to *kita* receptor expression, including the pineal gland, tail bud, and ear. In contrast, *kitlb* mRNA is expressed in brain ventricles, ear, and cardinal vein plexus, in regions generally not complementary to either zebrafish *kit* receptor ortholog. However, like *kitla, kitlb* is expressed in the skin during stages consistent with melanocyte survival. Thus, it appears that *kita* and *kitla* have maintained congruent expression patterns, while *kitb* and *kitlb* have evolved divergent expression patterns. We demonstrate the interaction of *kita* and *kitla* by morpholino knockdown analysis. *kitla* morphants, but not *kitlb* morphants, phenocopy the null allele of *kita,* with defects for both melanocyte migration and survival. Furthermore, *kitla* morpholino, but not *kitlb* morpholino, interacts genetically with a sensitized allele of *kita,* confirming that *kitla* is the functional ligand to *kita.* Last, we examine *kitla* overexpression in embryos, which results in hyperpigmentation caused by an increase in the number and size of melanocytes. This hyperpigmentation is dependent on *kita* function. We conclude that following genome duplication, *kita* and *kitla* have maintained their receptor–ligand relationship, coevolved complementary expression patterns, and that functional analysis reveals that most or all of the *kita* receptor's function in the embryo are promoted by its interaction with *kitla.*

## Author Summary

Gene duplication events provide a useful substrate to identify the effects of evolution in reshaping genes and their roles in physiology or development. Thus, dozens of receptor tyrosine kinases, with differing roles in development, have been generated in animal lineages. Less clear are how their associated ligands have duplicated and evolved and whether their evolution is constrained to match that of their cognate receptors. This report demonstrates the duplication of the *kit ligand* gene in zebrafish and shows that expression and function specific to the development of the melanocyte have been partitioned to one of these ligands, *kitla.* By this means, *kitla* coevolved with one of the duplicates of the *kit* receptor tyrosine kinase to regulate zebrafish melanocyte development. In contrast, the expression pattern of the other ligand, *kitlb,* which together with that of *kitla* approximates the expression of the mouse *kit ligand* gene, has evolved independently of either *kit* receptor gene.

## Introduction

Vertebrate melanocyte pigment patterns have long been studied as a model for pattern formation and morphogenesis. Melanocytes arise in the neural crest and immediately begin to migrate ventrally along distinct migratory pathways to establish the embryonic pigment pattern. Mutations in genes required for melanocyte morphogenesis have increased our understandings of cell–cell communication, signal transduction, differentiation, and cell migration. Mice with null mutations in the receptor tyrosine kinase *Kit (Dominant spotting, W)* are embryonic lethal as homozygotes due to the failure of hematopoiesis [1]. Animals heterozygous for null or homozygous for hypomorphic alleles are viable, however, and display a white spotting coat color phenotype. Further analysis of such mutants revealed an early failure in the melanocyte lineage: fewer melanoblasts arise in the neural crest, have defects in ventral dispersion, and then die before melanization [2–4]. The locus encoding the ligand for this receptor, *Kitl (Steel, Sl),* displays nearly identical deficiencies [5–8].

More recently, the use of the zebrafish, *Danio rerio,* has been a successful extension to our understanding of vertebrate pigment pattern biology. The optical clarity of zebrafish embryos, oviparous development, and genetic mutations in neural crest derived pigment cells make the zebrafish an attractive model for analysis of genetic mechanisms of pigment cell development. Additionally, the zebrafish genome is thought to have duplicated early in the teleost lineage [9–12]. Since then, most redundant genes have been lost, but for a small percentage both duplicates have been retained, presumably each evolving independently. One such co-orthologous pair is the zebrafish *kit* genes [13,14]. The zebrafish mutant for one of these, *kita (sparse),* has defects in embryonic melanocyte development; however, there are several differences from the role of *Kit* in mice. In contrast to mouse, zebrafish homozygous for *kita^b5^* (null) are viable and have no deficiencies in primordial germ cells or hematopoiesis. Zebrafish *kita* mutants retain 60% of wild-type embryonic melanocyte numbers [13]. These melanocytes fail to migrate to the periphery of the embryo and remain in dorsal regions along the neural tube and behind the otocysts. After 4 days postfertilization (dpf), these melanocytes undergo programmed cell death and are completely absent by 12 dpf. The second *kit* ortholog, *kitb,* is expressed in Rohon-Beard neurons, in trigeminal ganglion, and in the ear and does not seem to be involved in melanocyte development [14].

The primary roles of *kita* in zebrafish melanocyte migration and survival are separable. Genetic analysis of the receptor using a temperature-sensitive allele of *kita (kita^ jle99^)* revealed that *kita* signaling is required for migration before 2 dpf and then again is required for cell survival after this stage [15]. Furthermore, the identification of migration-specific and survival-specific alleles in *kita* suggests that these two functions may involve separate molecular domains of *kita* [15]. Although the migration-specific alleles all contained lesions near the putative ligand-binding domain, the allele that was defective for melanocyte survival had a lesion in the intracellular kinase domain of *kita*. One possible explanation for this finding is that survival is ligand independent in zebrafish.

Further evidence that *Kitl* and *Kit* have separable functions in migration and survival is revealed by mice mutant for *Neurofibromin* that have sustained RAS pathway activity [16]. These mice have melanocytes that require *Kit* signaling for migration but are independent of *Kit* signaling for melanocyte survival, indicating that activating the RAS pathway is sufficient for melanocyte survival but not migration in the mouse.

Separable functions for migration and survival are also established in the mouse for the *Kit* ligand, *Kitl* (also referred to as mast cell growth factor *[Mgf],* steel factor *[SlF],* and stem cell factor *[Scf]*). *Kitl* in mouse is expressed on the cell surface as one of two splice variants. The larger variant, *Kitl-1,* contains a primary cleavage site encoded in exon 6 that is rapidly cleaved at the membrane. In vitro experiments have shown that cells expressing this variant produce a soluble ligand product [17]. The *Kitl-2* isoform splices out exon 6, and although it does contain a secondary cleavage site in exon 7, its expression has been shown to be more restricted to the cell membrane than *Kitl-1* [17,18]. Furthermore, genetic analysis of several alleles of *Kitl* reveals that these splice forms may be involved in different functions of *Kit* signaling. Mice with the *Steel-dickie (Sl^d^)* mutation, which lack a transmembrane domain, produce only a soluble form of *Kitl* similar to that of *Kitl-1* [15,16] and have melanocytes that migrate properly but subsequently die [17]. This indicates a survival requirement for properly expressed *Kitl* on the basolateral surface of the epidermis. Transgenic mice expressing only *Kitl* lacking exon 6, however, appear to have wild-type pigmentation as adults and may not have a qualitative migration deficiency [18]. The current hypothesis is that a soluble ligand product is required to establish a chemotactic or chemokinetic gradient for promoting cell migration, whereas cell survival may be sustained through cell–cell contact via a membrane-tethered ligand. The alternative splicing of the primary cleavage site in exon 6 of *Kitl* may contribute to the regulation of this switch in development.

In this investigation, we show that the ortholog of tetrapod *Kitl* has duplicated in the zebrafish genome and the two co-orthologs have undergone splice form degeneration. One co-ortholog, *kitla,* has retained exon 6, while *kitlb* appears to have lost this exon. Given the suggested roles of each mouse isoform, we hypothesized that *kitla,* having retained exon 6, would be better suited to promote melanocyte migration, similar to mouse *Kitl-1,* while *kitlb*, having lost exon 6, would be required for melanocyte survival, like mouse *Kitl-2.* We test this hypothesis by first examining the mRNA expression of each gene using in situ hybridization and then by targeting antisense morpholino oligonucleotides (MOs) to each zebrafish co-ortholog of *Kitl*.

We show that *kitla* is expressed in the trunk along the medial melanocyte migration pathway during early migratory stages, 18 to 30 hours postfertilization (hpf), and then later expressed in the skin during stages (4 to 5 dpf) that require *kita* receptor function for survival. MO knockdown of *kitla* confirms that it is required for both melanocyte migration and survival. We also show that overexpression of *kitla* results in a hyperpigmented embryo with an increase in the number and size of melanocytes. A further demonstration that *kitla* is acting via the *kita* pathway is achieved through a genetic interaction between a low dose of *kitla* MOs and a sensitized allele of the *kita* receptor, and also by revealing that the hyperpigmentation phenotype of *kitla* requires *kita* function.

In contrast, *kitlb* is expressed in cells lining the brain ventricles, in the sensory epithelium of the ear, and in the cardinal vein plexus but not in regions associated with trunk melanocyte migration. However, like *kitla, kitlb* is expressed in the skin of 4 to 5 dpf larvae. MO knockdowns of *kitlb* indicate that it is not required for either embryonic melanocyte migration or survival. Finally, we discuss the possible signaling between the two *kit ligands* and *kit* receptors in zebrafish and the partitioning of functions after gene duplication events for receptor–ligand pairs.

## Results

### Identifying the Zebrafish *kit ligand* Orthologs

**Figure 1:**
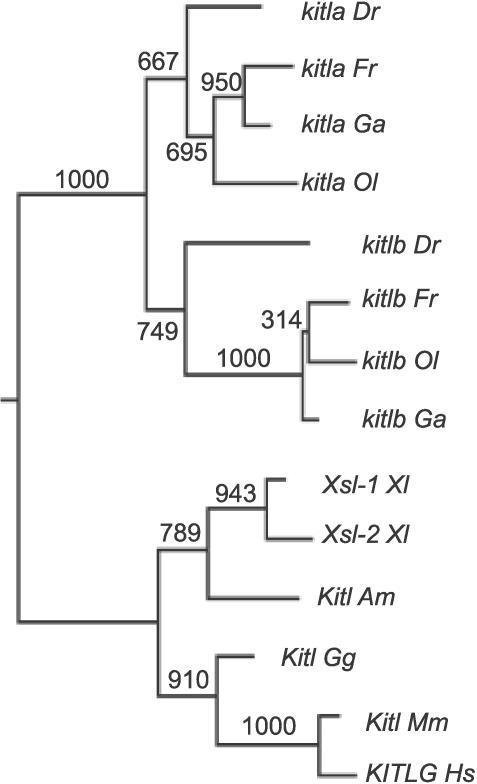
Phylogenetic Tree of *kitl* Protein Sequences The topology of the tree suggests that the *kitl* locus duplicated before the teleost radiation. *Dr, Danio rerio* (zebrafish), *Fr, Fugu rubripes* (fugu), *Ga, Gasterosteus aculeatus* (stickleback), *Ol, Oryzias latipes* (medaka), *Am, Ambystoma mexicanum* (axolotl), *Gg, Gallus gallus* (chick), *Xl, Xenopus laevis (Xenopus), Mm, Mus Musculus* (mouse), *Hs, Homo sapiens* (human)*.* Bootstrap values from 1,000 replicates are labeled.

We were interested in whether the *kita*-dependent pigment pattern in larval zebrafish required *kit ligand* (*Kitl* in mouse, *KITLG* in human, also referred to as *Mgf, Scf*). After searching the zebrafish genetic trace database for a *Kitl* ortholog using protein sequence from multiple species, we identified two partial candidate genes on Chromosomes 25 and 4, which we refer to as *kitla* and *kitlb,* respectively. Each candidate locus was amplified from 24-hpf embryonic cDNA to confirm the annotated sequence and to test whether each was expressed. Full-length cDNA was obtained for each candidate by 5′ and 3′ rapid amplification of cDNA ends (RACE) and by amplifying between exons. To test whether either of these candidates was orthologous to mammalian *Kitl,* we constructed a phylogenetic tree with several known tetrapod sequences, including mouse, human, chick, two copies in *Xenopus,* and axolotl (Figure 1). We were also able to identify two candidate copies of predicted *kitl* in the fugu, medaka, and stickleback genomes. Since the two copies present in the fish genomes are more closely related than either is to the tetrapod clade, it appears that the two zebrafish copies arose from a duplication event in the teleost lineage after the divergence from the tetrapod lineage but before the teleost radiation. This observation is consistent with the notion of a whole genome duplication in the teleost lineage at this time [9–11]. We conclude that zebrafish *kitla* and *kitlb* are co-orthologous to mammalian *Kitl*.

### *kitl* Co-orthologs Correspond to Different Mammalian Kitl Isoforms

**Figure 2:**
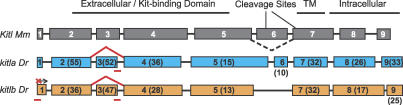
Genomic Structure Alignment with Mouse *Kitl* Exons (boxes) are drawn to scale and are labeled according to their homology with mouse sequence. Pairwise similarity (percent identical residues plus conserved amino acid substitutions based on the Blossum40 matrix) of the zebrafish protein to the mouse protein is presented for each exon in parentheses. Full-length values for mouse to zebrafish *kitla:* 29% identical and 43% similar. Full-length values for mouse to zebrafish *kitlb:* 20% identical and 50% similar. Although quite diverged in sequence, the zebrafish paralogs display well-conserved intron site locations with themselves and with the mouse ortholog. The best alignment of *kitlb* reveals that it has lost exon 6, which is alternatively spliced in mouse and human. Exon 5 of *kitla* has expanded 3′ with a corresponding contraction of exon 6. *Kit* binding domain contains the residues that interact with *Kit* as determined from crystal structures of the two mouse proteins [26,27]. The locations of the major cleavage site in mouse exon 6 and the minor cleavage site in mouse exon 7 are indicated. We cannot identify either cleavage site by sequence conservation in either zebrafish gene (see also Dataset S1). MOs (red bars) were targeted to overlap the ATG start site for *kitlb* and the exon 3–intron 3 boundary of *kitla* and *kitlb.* The splice site MOs resulted in splicing of exon 2 to exon 4 (red lines), resulting in a shorter, in-frame, transcript (see Figure 4E and 4F).

Because different mammalian *Kitl* functions have been shown to correspond with alternative splicing of exon 6, we examined the genetic structure and splice forms of zebrafish *kitla* and *kitlb.* To test whether zebrafish *kitla* and *kitlb* were alternatively spliced at exon 6 during larval pigment development, RT-PCR was conducted on each gene with primers flanking exon 6 from larva from 1, 2, 3, 4, and 5 dpf. Each gene displayed a single splice form (unpublished data). We then aligned the complete cDNA coding sequence of *kitla* and *kitlb* with the genomic sequence to reveal the exon–intron structure. A comparison with the genomic structure of mouse *Kitl* reveals that *kitla* contains nine exons, each aligning with the nine murine exons (Figure 2). In contrast, *kitlb* has retained only eight exons, with exons 1 through 5 aligning with murine exons 1 through 5 and exons 6 through 8 aligning with murine exons 7 through 9, suggesting that *kitlb* is missing sequence homologous to mouse exon 6 (Figure 2). Thus, duplication of the *kit ligand* gene in zebrafish was followed by splice form degeneration similar to what has been shown in other zebrafish duplicated genes [19].

**Figure 4:**
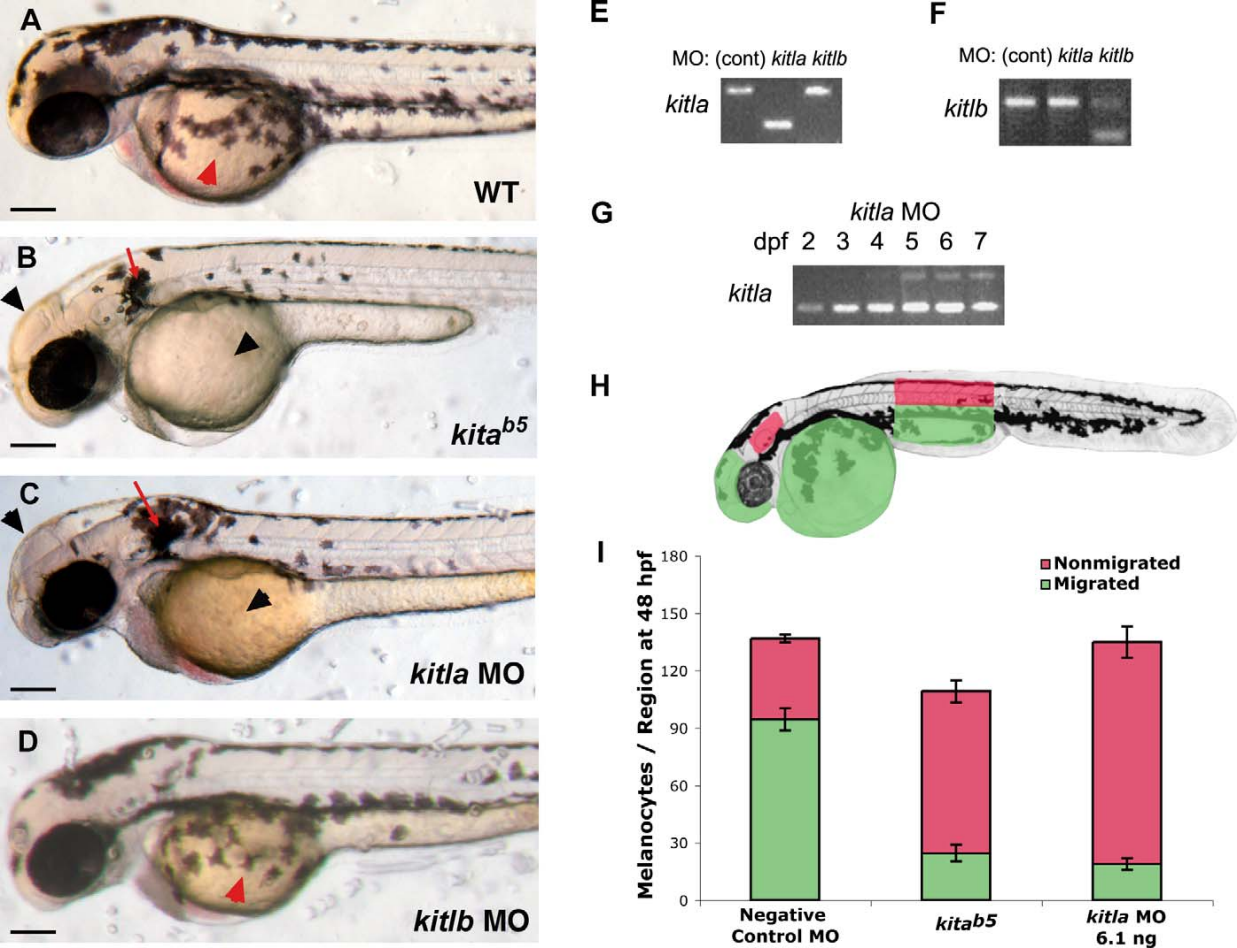
*kitla* Morphant Phenocopies *kita^b5^* Migration (A) Wild-type embryonic pigment pattern at 2 dpf shows melanocytes migrating over the yolk (red arrowhead). (B) *kita^b5^* mutants show migration phenotype, with melanocytes remaining near ear (red arrow) and dorsum and absent on yolk and head (black arrowheads). (C) Wild-type embryos injected with *kitla* MOs (6.1 ng) exhibit migratory phenotype similar to *kita^b5^* with melanocytes present near the ear (red arrow) and absent at the head and yolk (black arrowheads). (D) Wild-type embryos injected with *kitlb* MOs (6.0 ng) are indistinguishable from wild-type showing melanocytes present over the yolk (red arrowhead) by 2 dpf. (E–G) RT-PCR of morphant embryos shows MO specificity: (E) *kitla* RT-PCR of wild-type, *kitla* MO, and *kitlb* MO at 3 dpf; (F) *kitlb* RT-PCR of wild-type, *kitla* MO, and *kitlb* MO at 3 dpf; and (G) *kitla* RT-PCR of *kitla* MO at 2, 3, 4, 5, 6, and 7 dpf, revealing that aberrant splice product caused by the MO is dominant until 5 dpf, when wild-type message is visible. (H) Regions in embryo that were used to define migrated and nonmigrated melanocytes for quantitative analysis of melanocyte migration. Red areas indicate nonmigrated melanocytes in the dorsal and lateral stripe above the hind yolk and behind the ear. Green areas define migrated melanocytes on the head, on the yolk, and in the ventral and yolk sac stripe of the hind yolk. Note that melanocytes that have migrated to positions between the dorsum and the horizontal myoseptum, a region with typically no melanocytes, would be scored as nonmigrated in the embryo, while any melanocyte that migrates past the horizontal myoseptum would be scored as migrated, whether its migration is appropriate or not. (I) Quantitative analysis for melanocyte migration of negative control MOs (6.8 ng), *kita^b5^,* and *kitla* MO (6.1 ng). *kitla* MO embryos display a similar loss of migration as *kita^b5^*. Mean values with 95% confidence interval are reported, *n* = 10. Scale bars: 150 μm.

The presence or absence of exon 6 is not always associated with *kitla,* or *kitlb*, respectively, in the teleost. Both stickleback and fugu sequence defined as *kitla* in the phylogenetic tree are predicted to contain exon 6, while *kitlb* in both species does not. However, the predicted sequence for medaka *kitlb* appears to contain a candidate exon 6. This could indicate that the splice form degeneration of exon 6 occurred independently in these taxa, after the radiation of the teleost clade. However, it is possible that this exon is falsely identified by the gene-finding algorithm. Whether medaka *kitla* retains exon 6 is not clear, since the predicted transcript terminates at exon 5. In contrast to the zebrafish transcripts that are confirmed by RT-PCR experiments, the fugu, stickleback, and medaka sequences are only predicted GENSCAN sequences (http://genscanw.biosino.org or http://genes.mit.edu/GENSCAN).

The finding that zebrafish *kitla* and *kitlb* appear to resemble the mammalian alternative splice forms compelled the notion that these genes may be functioning similarly to roles suggested for the mouse isoforms. In mouse, alternative splicing appears to regulate the efficiency by which *Kitl* is cleaved from the cell membrane, as the isoform with exon 6 *(Kitl-1)* contains a primary cleavage site that is rapidly cleaved from the cell membrane [17]. This site has been identified (amino acid sequence VAAS) in mouse and is conserved in human and chick but unrecognizable in *Xenopus* and axolotl [20,21]. We next examined the protein sequences of the zebrafish genes for possible cleavage sites. We could not identify this cleavage site in zebrafish *kitla* exon 6 (see Dataset S1 for multiple sequence alignment). We did, however, notice a similar site in fugu (amino acid sequence VISS) in exon 6. In addition, the secondary cleavage site, which is present in both mouse isoforms in exon 7 and is cleaved at a lower efficiency, is not conserved in these species. Therefore, it is difficult to predict whether zebrafish *kitla* or *kitlb* is cleaved at the membrane and by what comparable efficiency.

The existence of two copies of *kit ligand,* each corresponding to an alternative splice form of mouse and human, lead us to hypothesize that zebrafish *kitla* retains the function of the mammalian isoform 1 and that *kitlb,* having lost exon 6, would retain the function of mammalian isoform 2. Thus, we predicted that *kitla* would be required for melanocyte migration, while *kitlb* would be required for melanocyte survival. These ideas were explored by examining the expression patterns and MO knockdown phenotypes of the zebrafish *kitl* co-orthologs.

### *kitla* and *kitlb* Expression Patterns

**Figure 3:**
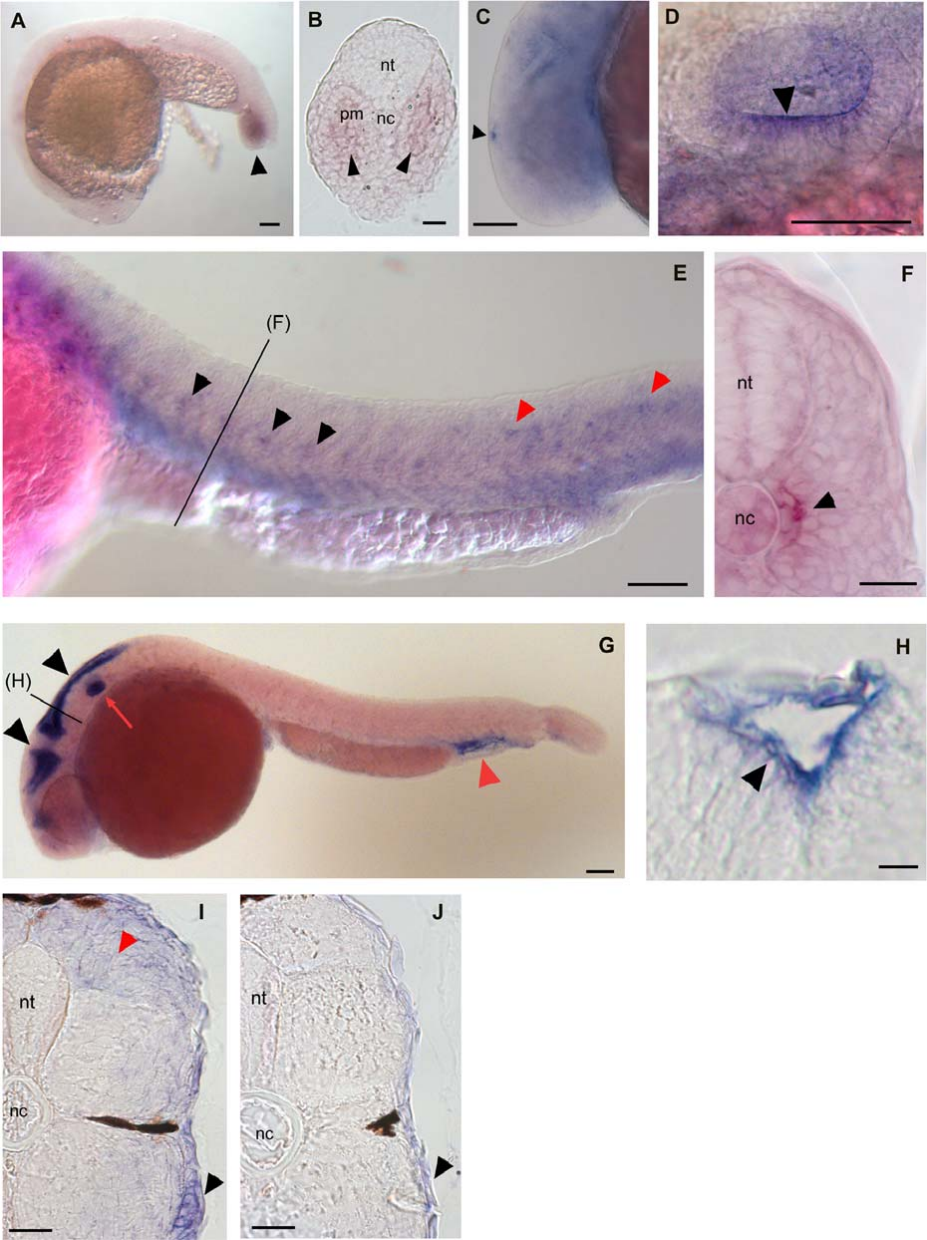
*kitla* and *kitlb* Whole Mount In Situ Hybridizations (A–F) *kitla* mRNA expression during stages of migration. (A) *kitla* expression is first seen at 19 hpf in the presomitic mesoderm of the tail bud (black arrowhead). (B) Section of 19-hpf tail bud. (C) High magnification shows expression of *kitla* mRNA in the pineal gland at 26 hpf. (D) High magnification shows *kitla* mRNA in the ear at 26 hpf in the sensory epithelium, with pronounced staining in the ventral otic vesicle (black arrowhead). (E) *kitla* mRNA is expressed in groups of cells at the horizontal myoseptum in the middle of each somite (black arrowheads) in the trunk beginning at 22 hpf through 30 hpf (image is 26 hpf). We also observe *kitla-*positive cells in more dorsal locations in the posterior somites (red arrowheads). (F) Cross section of trunk shows expression near notochord at 26 hpf. (G and H) *kitlb* mRNA expression during stages of migration. *kitlb* is expressed in the ventricles of the brain (black arrowheads), in the ear (red arrow), and in the cardinal vein plexus (red arrowhead) at 24 hpf (G). Cross section of brain ventricle shows *kitlb* expressed in cells lining the brain ventricles (H). (I and J) *kitla* and *kitlb* mRNA expression at 4 dpf, during stage of *kita*-dependent survival. *kitla* mRNA is expressed throughout the skin (black arrowhead) and in the dorsal myotome (red arrowhead) (I). *kitlb* mRNA is expressed faintly in the skin (black arrowhead) (J). Scale bars: (A) 100 μm, (B) 25 μm, (C) 100 μm, (D) 50 μm, (E) 100 μm, (F) 25 μm, (G) 100 μm, (H) 10 μm, (I) 25 μm, and (J) 25 μm. nt, neural tube; nc, notochord; pm, presomitic mesoderm

To explore whether either *kitla* or *kitlb* is involved in establishing the embryonic pigment pattern, we further examined their mRNA expression patterns during embryonic development using whole mount in situ hybridization (Figure 3). In mouse, *Kitl* mRNA expression is first found in the trunk at embryonic day 10.5 at the dorsal somitic dermatome [22,23]. This expression is lateral to the neural crest and is present at approximately the same time that *kita* mRNA*-*positive melanocyte precursors begin to migrate. Later, *Kitl* is expressed in the ectoderm, to support melanocytes after their migration to the skin [23].

Consistent with this role for *kitla,* we observe *kitla* mRNA in two groups of cells in every somite in the trunk from 20 hpf to 30 hpf (Figure 3E). Whole mount in situ analysis shows that the *kitla*-expressing cells appear in the center of each somite, and sections reveal that the cells are adjacent to the notochord (Figure 3F). It is interesting that *kitla* mRNA expression may be restricted to the middle of each somite; earlier observations of melanocyte migration showed that melanocytes appear to funnel to the middle of each somite as they migrate along the medial pathway [24]. That *kitla* mRNA expression overlaps with locations of melanocyte migration through the medial pathway supports the notion that *kitla* is involved in promoting the migration of melanocytes along this pathway. We cannot distinguish expression levels higher than background in regions along the lateral migration pathway during the appropriate stages (18 to 30 hpf). Thus, we find no evidence that a change in *kitla* expression is responsible for the change in migration from the medial to the lateral pathway. Instead, it appears more likely that this specific migratory behavior is controlled by factors other than *kitla.* We also examined whether *kitla* expression is present after 4 dpf, when *kita* is required for melanocyte survival. By 4 to 5 dpf, *kitla* expression has been lost in the medial trunk regions and is evident in the skin (Figure 3I). This expression is not restricted to melanocyte stripe regions but occurs throughout the skin, including the interstripe regions (Figure 3I, black arrowhead). We also see faint staining in dorsal regions of the somite (Figure 3I, red arrowhead). We suggest that the early expression from medial cells supports both medial and lateral migration and that the later switch to expression in the skin provides subsequent cues for melanocyte survival.

In addition to expression patterns of *kitla* suggestive of roles for pigment pattern, we see expression in places that may have no role in melanocyte development. Thus, we first observe *kitla* mRNA expression at the tail bud in 18 hpf embryos in the presomitic mesoderm (Figure 3A and 3B) as well as in the pineal gland of the 24-h embryo (Figure 3C). These regions are complementary to *kita* expression in the developing caudal notochord and pineal gland [13], suggesting a possible role in communication between the presomitic mesoderm (expressing the ligand, *kitla*) and the notochord (expressing the receptor, *kita*) and within the pineal gland (expressing both ligand and receptor). However, neither the *kita* mutant nor *kitla* morphants, as we describe below, display tail-bud or notochord-related phenotypes. *kitla* mRNA is also expressed in the sensory epithelium of the ear, most prominently in the ventral otic vesicle (Figure 3D, arrowhead). Interestingly, this otic expression is very near where the second zebrafish *kit* receptor, *kitb,* is expressed [14]. This suggests that there may be a functional communicative role conserved between *kitla* and *kitb,* although we can see no morphological phenotype in the ear of *kitla* morphants.

In contrast to *kitla* expression, *kitlb* is not expressed in regions that suggest a role in promoting melanocyte migration in the trunk. We find that *kitlb* mRNA is expressed in the ventricular proliferation zones, or pallium, of the brain by 22 hpf (Figure 3G and 3H, black arrowheads). This region has been described previously as a proliferative zone for neurons and glial cells [25]. As with *kitla, kitlb* expression was also detected in the ear (Figure 3G, red arrow). However, unlike *kitla,* for which sections show expresssion is limited to ventral otic vesicle, sections show that *kitlb* expression is found throughout the otic vesicle epithelium (unpublished data). We also observe less robust expression, in roughly half of embryos, of *kitlb* in the cardinal vein plexus by 26 hpf (Figure 3G, red arrowhead). These expression results suggest that *kitlb* is not expressed in locations that would seem appropriate for signaling to melanocytes during migration in either the medial or lateral pathway in the trunk. However, at 4 dpf, *kitlb* mRNA expression is evident in the skin (Figure 3J). *kitlb* expression at this stage appears less intense than *kitla* in the skin at these stages. These results are consistent with roles for *kitla, kitlb,* or both in melanocyte survival.

### *kitla* Is Required for Melanocyte Migration and Survival

To further test whether *kitla* or *kitlb* provides the ligand function for *kita* in melanocyte development, we explored their roles during embryonic development through MO gene knockdown (Figure 4). Our finding that the duplicated zebrafish *kitla* and *kitlb* resembled the alternative splice forms of mammalian *Kitl* and the foregoing analysis that *kitlb* expression is consistent with a role in melanocyte survival, while *kitla* expression is consistent with migration or survival, compelled the possibility that each gene corresponded functionally to the different mammalian splice forms. To further test the hypothesis that *kitla* and *kitlb* are required for melanocyte migration or survival, respectively, we designed splice-blocking MOs targeting the E3I3 exon-intron boundary specific to each gene and injected them into wild-type embryos. We chose this boundary because it is in the *kit*-binding domain [26,27] and is in the most conserved region in the multiple protein sequence alignment but different enough in the DNA sequence to confer specificity. RT-PCR analysis of the MO-injected embryos showed that the MO to each gene caused aberrant splicing, joining exon 2 to exon 4; thus each morphant splices out exon 3 of the targeted gene (Figure 4E for *kitla* and Figure 4F for *kitlb*). Sequencing the PCR product confirmed this splice product and results in an in-frame deletion of exon 3 (amino acids 41 through 59 for both *kitla* and *kitlb*). Furthermore, this result is specific, as the *kitla* MOs did not interfere with *kitlb* splicing at exon 3 and, conversely, the *kitlb* MOs did not interfere with *kitla* splicing (Figure 4E for *kitla* and Figure 4F for *kitlb*).

We next examined the consequences of MO knockdown on zebrafish embryonic melanocyte development. Melanocytes in wild-type embryos begin to migrate from the neural crest at 18 hpf and by 2 dpf establish the dorsal, lateral, ventral, and yolk sac stripes (Figure 4A). In contrast, melanocytes in *kita^b5^* mutants fail to migrate and remain near the ear and along the dorsal stripe, with very few appearing in the ventral stripe or yolk sac stripe (Figure 4B). Embryos injected with *kitla* MOs (6 ng) have a similar phenotype to *kita^b5^* (Figure 4C). Melanocyte counts at the regions outlined in Figure 4H reveal significantly fewer migrated melanocytes than wild-type, similar to *kita^b5^* (Figure 4I)*.* Interestingly, more melanocytes remain in the dorsal regions and near the ear than do in *kita^b5^* animals, resulting in a total melanocyte number similar to that of wild-type. However, we find that slightly higher doses of *kitla* MOs result in the same total number of melanocytes as for *kita^b5^*. This could indicate that the migration phenotype of *kitla* is more sensitive to abrogation than is the melanocyte number phenotype.

**Figure 5:**
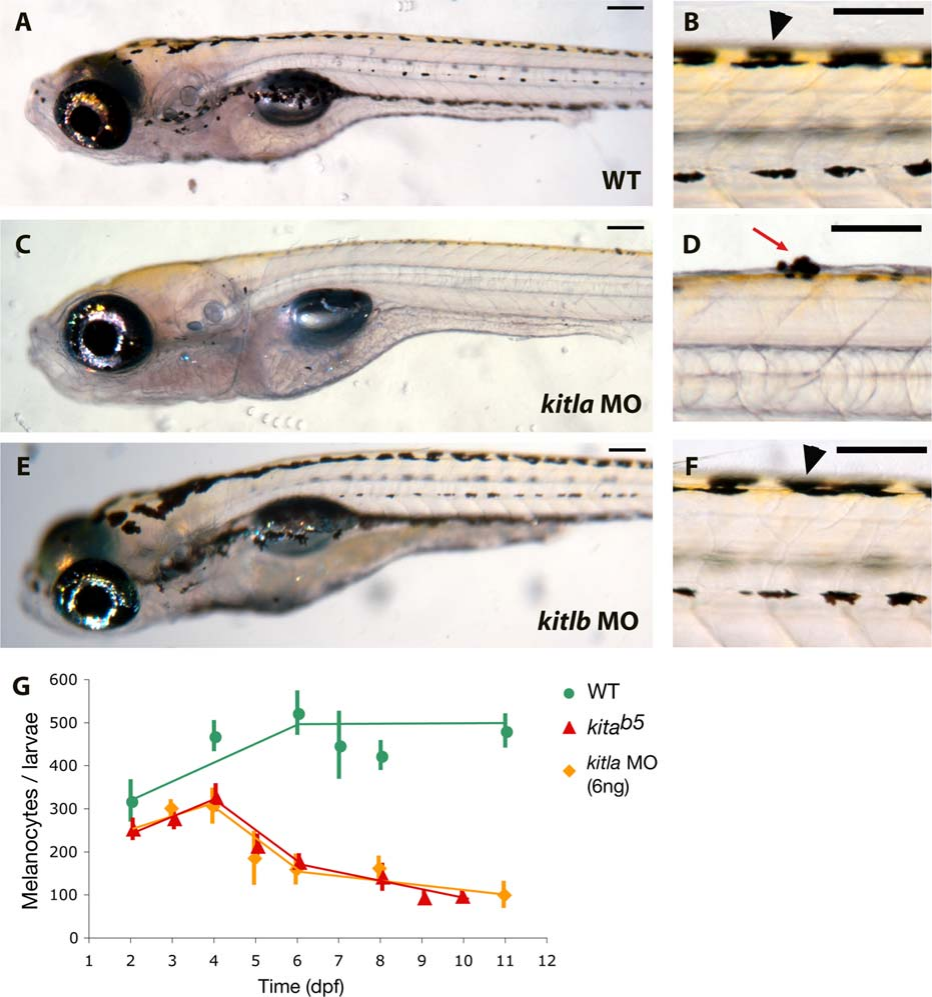
*kitla* Morphant Phenocopies *kita^b5^* Survival Phenotype (A) Wild-type larva at 8 dpf. (B) Higher magnification of dorsal melanocyte stripe of 8 dpf wild-type larva with healthy melanocytes (black arrowhead). (C) *kitla* MO shows fewer melanocytes at 8 dpf, similar to *kita^b5^.* (D) Higher magnification of *kitla* MO showing melanocytes blebbing through the skin (red arrow), characteristic of melanocyte programmed cell death. (E and F) *kitlb* MO at 8 dpf (E) and higher magnification (F) of *kitlb* MO 8 dpf, which is indistinguishable from wild-type. (G) Total melanocyte counts show that the survival phenotypes of *kita^b5^* and *kitla* MO are similar. Scale bars: 100 μm.

In addition to defects in migration, melanocytes in *kitla* MO embryos undergo programmed cell death in a manner similar to *kita*-null mutants (Figure 5). Wild-type larva have four distinct stripes at 8 dpf (Figure 5A) made up of healthy melanocytes (Figure 5B). Melanocytes in *kita* mutant larvae begin to die after 4 dpf, characteristically blebbing from the skin until the larvae are completely free of melanocytes after 12 dpf. Melanocytes of *kitla* morphants also begin to die after 4 dpf, have fewer melanocytes at 8 dpf (Figure 5C), and bleb from the skin as *kita* mutants (Figure 5D). Melanocyte counts reveal that *kitla* morphants have almost the same extent of melanocyte loss as do *kita* mutants (Figure 5G). Although usually all the melanocytes in *kita* mutants disappear, a very few melanocytes of *kitla* morphant embryos tend to survive. This is possibly due to partial restoration of *kitla* function, as MOs tend to lose efficacy over time [28,29]. We first see restoration of the wild-type *kitla* transcript at 3 dpf and increasing wild-type transcript through 7 dpf (Figure 4G), during the stages when melanocyte survival is *kita* dependent. These results indicate that *kitla* promotes both migration and survival in melanocytes similar to the zebrafish *kita* receptor.

### *kitla* MOs Enhance *kita^jle99^*

**Figure 6:**
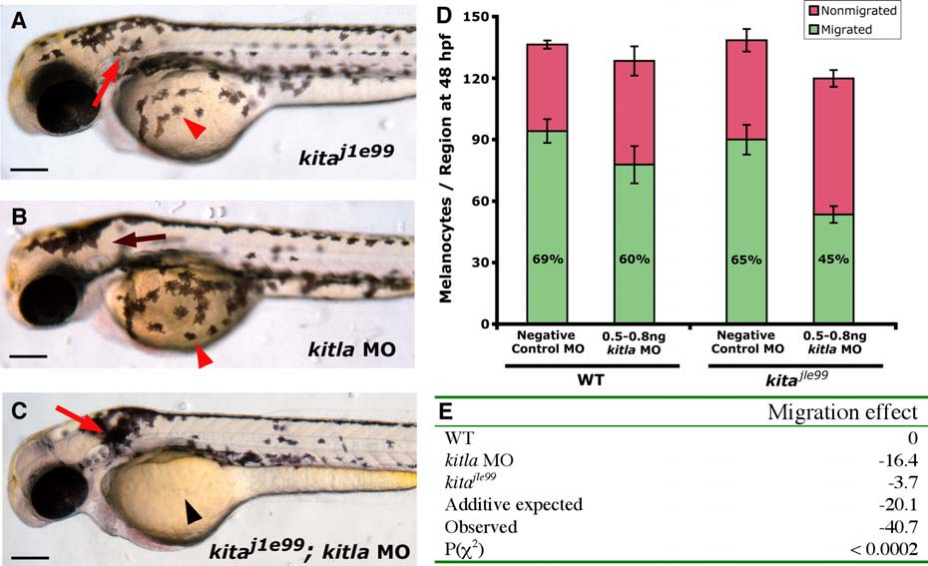
*kitla* MO Enhances Temperature-Sensitive *kit^j1e99^* Allele (A) *kit^j1e99^* embryos reared at 28 °C appear similar to wild-type melanocyte pattern (see Figure 4A for wild-type) at 3 dpf. (B) A submaximal dose (0.5 to 0.8 ng) of *kitla* MOs shows little effect in wild-type embryos. (C) Submaximal dose of *kitla* MOs shows a significant migration phenotype in *kit^j1e99^*. (D and E) Quantitative analysis of *kit^j1e99^*–*kitla* enhancement. Melanocytes were counted at 3 dpf and scored as migrated if in green regions or nonmigrated if in regions shown in red (Figure 4H). Mean values with 95% confidence interval are reported (*n* = 10). (D) Using this metric, wild-type embryos average 94.4 (69% of total) migrating melanocytes. A submaximal dose of *kitla* MOs results in 16.4 fewer migrated melanocytes than wild-type. *kit^j1e99^* embryos reared at 28 °C have 3.7 fewer migrated melanocytes. Neither the number of total melanocytes or of migrating melanocytes in *kit^j1e99^* nor the submaximal dose of *kitla* MOs is significantly different compared with wild-type. (E) Migration effect is defined as the difference in migrating melaocytes compared to wild-type. If the combined effects of  *kit^j1e99^* and the submaximal dose of *kitla* MOs are additive, we expect a migration effect of −20.1 in the *kit^j1e99^*–*kitla* MO larvae (−16.4 ± 3.7). Instead, we observe a migration effect of −40.7. The χ^2^ test between the number of migrated melanocytes in the *kit^j1e99^*–*kitla* MO larvae and the expected number reveals this difference to be significantly (*p* < 0.0002) greater than additive. Scale bars: 150 μm.

The phenotypic analysis of *kitla* MOs in wild-type embryos suggests that *kitla* may be functionally orthologous to mammalian *Kitl,* as the ligand for the *kita* receptor tyrosine kinase. To further test whether *kitla* is in the *kita* pathway, we investigated the genetic interaction between the effects of the *kitla* MOs and a sensitized *kita* allele (Figure 6). *kita^jle99^* is a temperature-sensitive allele of *kita*, with a lesion in the C-terminal tyrosine kinase domain (Leu754Pro) [15]. *kita^jle99^* fish are phenotypically wild-type at the permissive temperature (25 °C) but show defects for melanocyte migration and survival at the restrictive temperature (33 °C) similar to a null *kita* mutant. For our purposes, we take advantage of the intermediate phenotype at 28 °C (Figure 6A), in which slightly fewer melanocytes migrate compared to wild-type. This hypomorphic phenotype is likely due to partial *kita* signaling activity and may therefore be used as an assay for testing whether other genes act in the same genetic pathway as *kita*. Our analysis shows that the interaction between *kita^j1e99^* and *kitla* MOs is synergistic, with a much greater deficit in migrating melanocytes (−41.7) than either mutant alone (−3.7 melanocytes) or morphant alone (−16.4 melanocytes), or the expected additive deficit (−20.1 melanocytes) (Figure 6E). This result confirms a genetic interaction between *kita* and *kitla.*

### *kitla* Overexpression Induces Hyperpigmentation

**Figure 7:**
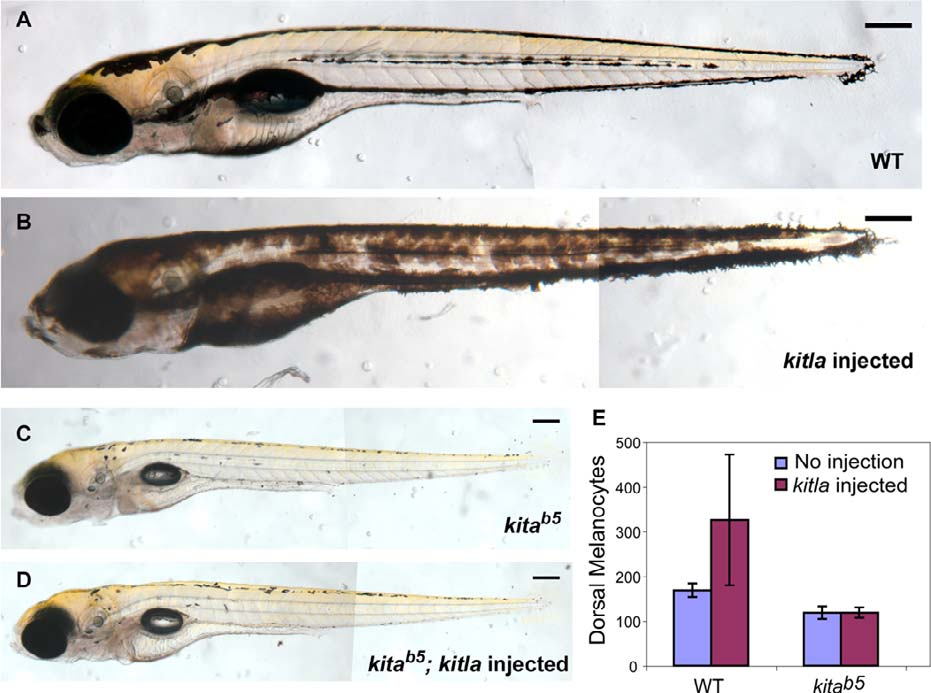
*kitla* Overexpression Causes Hyperpigmentation in Wild-type but Not in *kita^b5^* Embryos (A) Wild-type larva. (B) Larva injected with *kitla::JRed* fusion construct shows hyperpigmentation with melanocytes covering a larger area than wild-type. (C) *kita* larva. (D) *kitla::JRed* injected into *kit^b5^* embryos results in *kit^b5^* phenotype. (E) *kitla::JRed* hyperpigmented larvae have more melanocytes on the dorsal stripe than do wild-type. When *kitla* expression vector is injected into *kita* embryos, there is no change in the number of melanocytes. All larvae at 6 dpf (*n* = 10). Scale bars: 150 μm.

To further explore the role of *kitla* in establishing the melanocyte pattern, we examined *kitla* overexpression in wild-type embryos (Figure 7). Accordingly, we constructed an expression vector with a *kitla* fusion to the red fluorescent protein, JRed [30]. Injection of this vector, or capped mRNA from this vector, into wild-type embryos results in embryos with hyperpigmentation throughout the body (Figure 7B). To test whether this was due to an increase in the cell number, melanocytes were counted along the dorsal stripe. Wild-type embryos have an average of less than 200 dorsal melanocytes (Figure 7A and 7E). Hyperpigmented animals show a dramatic increase in the number of dorsal melanocytes, averaging over 300 cells or an increase of greater than 50% (Figure 7E). The number of melanocytes in other regions also appears increased in these embryos, although we have not quantified that increase.

In these transient injection experiments, we typically see little red fluorescence except in cells around the yolk. Because JRed and *kitla* are each expected to act as dimers [30,31], it is possible that, through steric hindrance, *kitla* dimerization interferes with the ability of JRed to dimerize correctly and therefore is not visible at all locations of expression. Whole mount in situ analysis shows abundant and highly mosaic mRNA expression (unpublished data), suggesting that *kitla* protein is expressed throughout the embryo in these experiments.

### *kitla-*Induced Hyperpigmentation Is *kita* Dependent

To firmly show that *kitla* acts through the *kita* receptor, we explored the effect of overexpressing *kitla* in *kita^b5^* embryos (Figure 7). When the *kitla* expression construct is injected into *kita^b5^* embryos (Figure 7D), melanocytes develop identically to the *kita-*null phenotype (Figure 7C), including an identical number of melanocytes in the dorsal stripes (Figure 7E). This is consistent with the notion that the effects of ligand, *kitla,* are mediated only through the receptor, *kita.* Together with the phylogenetic analysis, the MO phenocopy of *kita^b5^,* the MO enhancement of *kita^j1e99^,* and the dependence on *kita* for the *kitla-*induced hyperpigmentation, we conclude that *kitla* is the functional *kita* ligand in zebrafish embryonic melanocyte development and that *kitla* promotes all of the *kita*-dependent functions in embryonic melanocyte development. The finding that *kitla* MO largely phenocopies the *kita* mutant tends to argue that *kitla* is the only ligand required for embryonic melanocyte development, although it is difficult to rule out a small effect for *kitlb* that is not revealed in our experiments.

### *kitlb* Is Not Required for Embryonic Melanocyte Patterning or Survival

The above demonstration that *kitla* is necessary for melanocyte migration and survival roles of the *kita* receptor does not preclude the possibility that *kitlb* also participates in this process. Indeed, our expression data at 4 dpf show that *kitlb* may be involved in melanocyte survival. When we injected *kitlb* E3I3 MOs to block normal splicing of *kitlb* resulting in most transcripts (approximately 75%) lacking exon 3 (Figure 4F), we found no effect on melanocyte phenotypes. *kitlb* morphant embryos are not significantly different from wild-type in melanocyte migration (Figure 4D) or survival (Figure 5E and 5F). Additionally, *kitlb* morphants appear morphologically normal and show no obvious blood defects or anemia at 2 dpf (unpublished data). Because we could not rule out the possibility that *kitlb* is still functional despite the ability of E3I3 MOs to splice out exon 3, we designed two additional *kitlb* MOs designed to block translation. Neither of these showed an effect on melanocyte migration or survival at all conditions tested. For a more sensitive test, we again took advantage of our conditional *kita* allele by injecting these *kitlb* MOs into embryos homozygous for *kita^jle99^,* to examine whether they could enhance the sensitized *kita* phenotype. We failed to detect an enhancement in *kita^jle99^* with these *kitlb* MOs, either independently or as a combined mix (unpublished data). Taken together, along with the expression of *kitlb* mRNA, these data suggest that *kitlb* does not have a necessary role in embryonic melanocyte development.

## Discussion

We have identified and explored the roles of two *kitl* orthologs in zebrafish. These co-orthologs are notable by their differences in genomic structure, expression pattern, and phenotypes from MO knockdown experiments.

It is clear that gene duplications have played a role in the creation of the large number of receptor tyrosine kinases [32,33]. Less clear is the role of gene duplication in generating their protein ligands, since they evolve more rapidly and the phylogenetic relationships are not as well characterized. However, at least in some instances, receptor–ligand pairs could have arisen through the duplication of both the receptor and ligand. Such may have been the case with *Kit/Kitl* and *Fms/M-Csf*. While *Kit* is clearly most closely related to *Fms* [34]*,* the protein sequences of *Kitl* and *M-Csf* are difficult to align. However, the secondary structures of these ligands indicate that, of all the short-chain helical cytokines, *M-Csf* is the most similar to *Kitl* [26]*.* The more recent duplication leading to *kita/kitb* and *kitla/kitlb* in zebrafish, therefore, provides an opportunity for understanding the evolutionary mechanisms for the coevolution of receptor–ligand pairs.

Both the *kit* receptor and its ligand appear to have duplicated in the teleost lineage. These duplication events are consistent with the notion of a whole genome duplication event prior to the teleost radiation [9–11]. It has been hypothesized that gene duplication events are often followed by relaxed evolutionary selection which can result in changes in expression or function of the co-orthologs [35]. Preservation of both copies may result through subfunctionalization, whereby multiple functions controlled by a single ancestral gene are partitioned between gene duplicates [36]. Subfunctionalization of gene expression has been described for several zebrafish co-ortholog pairs [37–39]. Similar to the case for *kitl* we describe here, the zebrafish *mitf* gene also duplicated, and each co-ortholog generates a transcript that corresponds to one of the splice forms produced by the mammalian *Mitf* gene [19,40].

### *kitla* Is the Functional Ligand for *kita* in Melanocyte Migration and Survival

Examination of the genetic structure at the *kitla* and *kitlb* loci revealed that a likely splice form degeneration followed the duplication event, where *kitla* retained exon 6 and the *kitlb* gene lost this exon. Interestingly, the presence or absence of exon 6 in the mouse transcript regulates the inclusion of a primary cleavage site that alters the soluble expression of the ligand [17,18]. There is evidence that the alternative splice forms, *Kitl-1,* containing both the primary and secondary cleavage sites, and *Kitl-2,* with only the secondary cleavage site*,* may be functioning for promoting melanocyte migration and survival, respectively [22]. It has also been shown, however, that exclusive expression of the short splice form, lacking exon 6, *Kitl-2,* results in a normal adult pigment pattern [18]. Indeed, both isoforms appear to be cleaved at the membrane [17], albeit at different rates, and differences in the functions of the two isoforms may be quantitative, rather than qualitative.

Since the zebrafish *kita* melanocytic functions appear to be molecularly separable, we explored the possibility that the migration and survival functions were partitioned between the zebrafish *kitl* co-orthologs. Although the primary cleavage site is conserved among many species, we were unable to identify such a site by protein sequence in any of the fish sequences we examined (see Dataset S1). This is not entirely surprising given the rate of evolution of these ligands. It is possible that such a site exists but is not well conserved.

Our analysis of the expression patterns, MO knockdown phenotypes, interaction of MO knockdown with a sensitized *kita* allele, and finding that overexpression phenotypes of *kitla* require *kita* function now argue that *kitla* provides all of the necessary ligand roles for the *kita* receptor in promoting embryonic melanocyte development, whereas *kitlb* seems to provide little or none*.* These roles include establishing the initial cell number, promoting migration, and promoting survival. Temperature shift experiments with the *kit^j1e99^* temperature-sensitive allele of *kit* revealed that melanocytes require *kit* signaling before 2 dpf for migration and after 2 dpf for survival [15]. The finding that *kitla* is required for both of these indicates that it is involved first to promote migration before 2 dpf and then again later to promote survival after 2 dpf. That both the migration and survival defect of the *kitl* MOs phenocopy those of *kita* null suggests that the difference in time for these functions is not due to differential expression or display of the ligand after 2 dpf. It is more likely that this switch is mediated downstream of *kita* signaling. We note that we have not explored the individual roles of *kitla* or *kitlb* in promoting adult melanocyte development, with its different *kita-*dependent and *kita-*independent melanocyte populations [41,42].

Previously, an exhaustive noncomplementation screen for additional alleles of *kita* resulted in several migration-specific alleles with lesions near the ligand-binding domain, while the single survival-specific allele had a lesion in the intracellular domain [15]. One possible explanation for these results was that only the migration-promoting function of *kita* required ligand interaction, and that the melanocyte survival function of *kita* might be ligand independent. The finding that the *kitla* MO abrogates both melanocyte migration and survival rules out that possibility. In light of our results here, it is more likely that the intracellular lesion may disrupt binding of downstream targets of *kita* that are survival specific. It also appears as though the separable functions of the *kitla*/*kita* pathway diverge at *kita*, and not by the activity of *kitla.*

### Coincident Gene Expression of *kit*–*kitl* Pairs

We were interested in how expressions of the two zebrafish *kitl* co-orthologs have evolved since their duplication event. In our analysis, we will assume that the *Kitl* expression patterns in mouse [22,23,43] and chick [43] are similar to that of the tetrapod/fish ancestral pattern and examine how these gene expression domains were partitioned to the zebrafish *Kitl* co-orthologs. Thus, *Kitl* is expressed in the trunk at the lateral and intermediate mesoderm in both mouse and chick [23,43,44] and in the dorsal dermomyotome in mouse [22]. *Kitl* is also weakly expressed throughout the brain and is expressed near the otic vesicle in the mesenchyme between the hindbrain and the surface ectoderm, with more pronounced expression in regions lateral and ventral to the otic vesicle [45].

**Figure 8:**
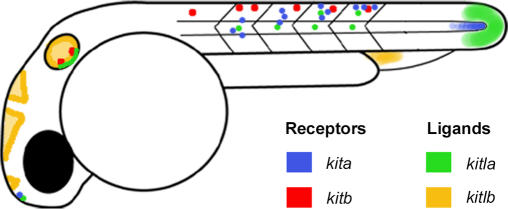
Stylized Drawing of Zebrafish Embryo Displaying mRNA Expression Patterns of *kita* and *kitb* Receptor Tyrosine Kinases and Their Candidate Ligands, *kitla* and *kitlb* Coincident expression is noted for *kita* (blue) and *kitla* (green) in the trunk, tail bud, ear, and pineal gland. *kitb* (red) and *kitlb* (yellow) do not appear coincident. *kitlb* does not appear to have a coincident receptor for its expression in brain ventricles or in cardinal vein plexus. *kitb* may be coincident with *kitla* expression in the trunk.

In composite, the early expression domains of the two *kitl* genes in zebrafish match much of that of mouse or chick, or that assumed for the ancestral form. These domains, together with the domains of the *kit* receptors, are shown in a stylized embryo (Figure 8) representing expression patterns seen from 18 to 30 hpf. Additionally, this diagram reveals the coincident expression of *kita* and *kitla,* consistant with *kitla-*positive cells signaling to cells expressing *kita*. Most important, *kita* is expressed in migrating melanocytes (Figure 8, blue shading along trunk) and *kitla* is expressed in cells along the medial pathway for melanocyte migration (green shading along trunk). Another location where *kita* and *kitla* are coincident is the tail bud, where *kita* is expressed in the notochord [13] (Figure 8, blue) and *kitla* is in the surrounding presomitic mesoderm (Figure 8, green shading at tail). Additionally, both *kitla* and *kita* are expressed in the pineal gland (Figure 8 *,* green/blue regions), suggesting that they may be interacting to promote some aspect of pineal gland function or development. Such involvement of the *kita* pathway does not appear to be required for normal development, since we do not observe morphological changes in these tissues in either *kitla* morphants or *kita^b5^* mutants. Moreover, like *kitla,* the expression of *kita* is a subset of the expression domains observed in mouse [13]. Thus, we conclude that coevolution of *kita* and *kitla* results in retained complementary expression patterns, following partition of the ancestral *kit* and *kitl* expression. These expression patterns suggest they share functional roles in embryonic development.

Next, we examined the possibility that expression patterns of *kitb* and *kitlb* have also coevolved with one another. We previously reported that the receptor *kitb* is expressed in Rohon-Beard neurons, trigeminal ganglion, and the otic vesicle [14] (Figure 8, red shading). With the possible exception of the otic vesicle, where we observe both *kitla* and *kitlb* expression, none of these locations appears to have coincident expression with *kitlb*. Like *kitla,* we find *kitlb* expressed in the epithelium lining the otic vesicle (Figure 8, yellow shading). It is interesting to note that in mouse, *Kitl* seems to be expressed in mesenchyme near the ear, rather than the otic vesicle epithelium, as we describe for *kitla* and *kitlb.* These expression domains are clearly not homologous, yet their similar positioning raises the possibility that they reflect homology of function. If so, it would be of interest to know how these expression domains evolved in different tissues. Since *kita* is not expressed in the ear, it remains possible that either or both ligands may be signaling to *kitb* in this context.

We also see locations of *kitlb* mRNA expression where there is no coincident *kita* or *kitb* receptor expression. These areas include the cardinal vein plexus and in the cells lining the ventricles of the brain (Figure 8, yellow shading). Although no obvious morphological phenotypes were seen in *kitlb* morphants, more sensitive assays, for instance, for behavior or blood histology, might reveal more subtle defects.

We conclude from the coincident mRNA expression patterns of *kitla* and *kita*, together with the similar phenotypes caused by mutation or MO knockdown, that *kitla* and *kita* coevolved expression patterns and functions, presumably by partitioning select expression domains and functions from the ancestral receptor and ligand genes. In contrast, except for the expression in the ear, there is little or no coincident expression of *kitlb* and *kitb* in the embryo. This raises the possibility that *kitlb* does not signal through the *kitb* receptor. By this logic, *kitlb* and *kitb* might interact with other receptors or ligands, perhaps related through more ancestral paralogs. It will now be interesting to know how the structures of *kitla* and *kita* coevolved to maintain effective ligand–receptor interactions and, in contrast, how *kitlb* and *kitb* structures evolved with their as-yet-unidentified receptor or ligand partners.

In addition to these early expression domains, both *kitla* and *kitlb* mRNAs are expressed at 4 to 5 dpf during *kita-*dependent melanocyte survival. At this stage, *kita-*expressing melanocytes have established the embryonic melanocyte pattern. However, both *kitla* (abundantly) and *kitlb* (weakly) are expressed throughout the skin and are not restricted to regions adjacent or near melanocyte stripes. The presence of *kitla* expression in the interstripe region of the skin indicates that although this gene is required (perhaps permissively) for migration, *kitla* expression does not establish a prepattern for melanocyte stripe formation.

The partitioning of expression domains between the zebrafish *kit/kitl* co-orthologs is the result of about 300 million years of evolution since the zebrafish genome duplicated [12]. We can compare this with the more recent duplication of *kitl* in *Xenopus (*called *Xsl-1* and *Xsl-2)* [21]. In contrast to the teleost, both *Xenopus* genes appear to contain exon 6 (see Dataset S1). Overall, the difference in the expression domains between *Xsl-1* and *Xsl-2* is less than what we see between zebrafish *kitla* and *kitlb.* Like zebrafish, both copies appear expressed in the otic placode and in the skin. Unlike zebrafish, both *Xenopus* genes are expressed in the dermomyotome of the trunk, with *Xsl-1* expressed throughout the length of the trunk and *Xsl-2* restricted to the anterior somites. It is less clear how these expression domains are functioning in *Xenopus* in relation to melanocyte development, as neither reported co-ortholog of *Kit* appears to be expressed in melanocytes [46,47].

### *kitla-*Induced Hyperpigmentation

As a first step toward understanding the morphological roles of *kitla* in melanocyte development, we have begun to generate animals that overexpress the ligand. Following injection of the *kitla* expression vector, embryos that overexpress *kitla* are hyperpigmented with more and larger melanocytes. The finding that this hyperpigmentation is dependent on *kita*, together with the *kitla* MO knockdown phenotype and its interaction with the sensitized temperature-sensitive *kita* allele, provides compelling evidence that *kitla* is the ligand for *kita* in embryonic melanocyte development. It would be interesting to know whether similar overexpression of *kitlb* also results in hyperpigmentation, which might shed light on whether *kitlb* still has the capacity to bind *kita* and transduce a signal.

As yet, it is unclear whether *kitla-*overexpressing embryos have more melanocytes because of proliferation of precursor cells, recruitment and differentiation of additional stem cells, transdifferentiation from nonmelanized cells to melanocytes, or protection from cell death. Regardless of the mechanism, this result suggests that in normal development, *kitla* is rate limiting, and the correct amount of expression is necessary to produce the wild-type embryonic pigment pattern. Notably absent in a variety of large-scale [48] and more focused [49,50] mutant screens in zebrafish are mutants with melanocyte hyperpigmentation, either by increased number of cells or larger cells. It is interesting that the phenotype produced by *kitla* DNA injection, although expected to produce a mosaic expression of *kitla*, has an effect throughout the entire animal. Our analysis to understand the role of *kitla* in melanocyte development is ongoing, and we are now making stable transgenic lines expressing this construct that will allow us to explore the role of *kitla/kita* signaling in cell number, in cell size control, and in directing melanocyte migration.

## Materials and Methods

### Fish rearing and stocks.

Embryos were obtained through in vitro fertilization and reared on a 14:10-h dark/light cycle in egg water at constant temperature of 28 °C, unless otherwise noted. Wild-type fish were obtained from the sjC inbred line or from sjA/sjC hybrids [51,52]. Mutants in the kit receptor tyrosine kinase, *kit^b5^* or *kit^j1e99^,* have been previously described [15].

### Cloning *kitla* and *kitlb.*

Candidate zebrafish *kitl* sequences were found by searching the Whole Genome Shotgun Trace Database (http://www.ncbi.nlm.nih.gov/Traces/trace.cgi?&cmd=retrieve&val=species_code%3D%22DANIO%20RERIO%22&retrieve=Submit; June 2002) using the tblastn program [43] with *Kitl* protein sequences from human, mouse, chick, *Xenopus,* and axolotl as the query. Each query sequence appeared to produce two conserved alignments in the zebrafish genome, with the most significant pair being against human *KITLG* exon 4, with E-values of 4e−9 and 1e−5. This pair of hits was mapped on the LN54 radiation hybrid panel to Chromosomes 25 and 4 and named *kitla* and *kitlb,* respectively.

To obtain full-length cDNA sequence of these loci, PCR primers were designed to amplify within and between these candidate *kitl* exons from embryonic and caudal fin poly(T)-primed cDNA. RACE was used to attain the 5′ and 3′ ends of the genes. In the case of *kitlb,* where RACE failed, primers were designed from candidate open reading frames downstream and upstream of the known exons in the genomic region (assembly z06s024825). Single RT-PCR fragments were then isolated from the reaction by 1% agarose gel electrophoresis and sequenced.

### Phylogenetic and sequence analyses.

A multiple sequence alignment of kit ligand protein sequences was generated with ClustalW and adjusted manually (see Dataset S1). Because the alignment of the protein sequence C-terminal to exon 5 was ambiguous, Kyte-Doolittle hydropathy plots and the hidden Markov modeling program HMMTOP [53] were used to confirm the alignment of the transmembrane domain of exon 7. Exons 6 through 9 were omitted from the phylogenetic analysis. Phylogenetic relationships were then determined using the maximum likelihood method with the Phylip package [54]. Bootstrap support for the topology was conducted with maximum likelihood with 1,000 replicates. We also built trees using the neighbor joining and parsimony methods. Neighbor joining produced a tree with zebrafish *kitla* as an outgroup of the other fish genes, while parsimony produced a tree with zebrafish *kitlb* as the outgroup. The maximum likelihood tree is presented because it is most consistent with the pattern of gene duplication previously reported in the teleost lineage and would require the least amount of evolutionary changes to explain the two copies in each fish species.

### In situ hybridization.

*kitla* and *kitlb* probe template was generated by PCR amplification from 24-hpf embryonic cDNA. Primers used to amplify *kitla* and *kitlb* cDNA were adapted with T7 RNA polymerase recognition sequences on the 3′ end of the coding sequence: *kitla* forward primer 5′-TCTCGTTCCATATGAAGAAGTCAA-3′, reverse primer 5′-taatacgactcactataggTCAGATATCCCCACATCTAATGG-3′, *kitlb* forward primer 5′-CACATGAGGGAGGTTAAGATTG-3′, and reverse primer 5′-taatacgactcactataggACACCCTAAAGAATCCAGCCA-3′, where lowercase letters indicate T7 sequence.

Antisense probe was then synthesized from the PCR products using DIG-labeled NTPs in a transcription reaction with T7 polymerase. The sense strand was also synthesized using T7 adaptors on the 5′ end of the coding sequenced and used as a negative control. Whole mount in situ hybridization was modified from previously published methods [55] for use with an Abimed in situ robot (Abimed In Situ Pro; Intavis AG, http://www.intavis.com). Color development was conducted manually.

For sectioning, embryos were embedded in agar [56], and 10-μm sections were cut using a Leica CM 1850 Cryostat (http://www.leica.com).

### MO knockdown analysis.

Antisense MOs (Gene Tools, http://www.gene-tools.com) were designed to reduce the amount of functional protein expressed in embryos [57]. We chose to target mRNA splicing in order to test the efficacy of the MOs through RT-PCR analysis [58]. The MOs *kitla* E3I3 (5′-CTGGATAACAACACTCACCACTTCT-3′) and *kitlb* E3I3 (5′-CACATGTATACTTACCACATCCTTT-3′) were designed to block processing at the splice donor site of exon 3. Additional MOs were designed to target the *kitlb* ATG start site (5′-ATGTCTTAACCTCCCTCATGGGGAACAT-3′) and 5′ UTR (5′-CCTGTAAATGACTGGAGATAATCAC-3′). Negative control MO (5′-CCTCTTACCTCAGTTACAATTTATA-3′) was used as a control for nonspecific, MO effects. MO injection solution was made up of 20 μM MO in 1× Danieau buffer with 1% Phenol Red as a visual tracer. Embryos were injected with MO injection solution in the yolk at the one- to two-cell stage.

To test efficacy of each MO in altering the transcript for *kitla* and *kitlb,* RT-PCR was conducted on embryos injected with *kitla* E3I3 MOs, *kitlb* E3I3 MOs, and negative control MO (Cont. MO). Primers for RT-PCR were *kitla* forward primer 5′-CACAGTTGCTGCCTATTCCA-3′, *kitla* reverse primer 5′-TGAATCCTCCAAACCAGGTC-3′, *kitlb* forward primer 5′-ACCTGCTCAGGTGTTTTTGG-3′, and *kitlb* reverse primer 5′-CATTCTGTCCTCCAGGTCGT-3′. A semiquantitative measure of *kitla* and *kitlb* MO efficacy was measured by a pixel intensity calculation in ImageJ (Research Services Branch, National Institute of Mental Health, http://www.rsb.info.nih.gov/ij).

### *kitla* overexpression.

*kitla* cDNA sequence was cloned in frame into the pJRed-N vector (Evrogen, http://www.evrogen.com) to produce a C-terminal fusion protein. This resulted in an expression vector with an early promoter of cytomegalovirus driving the expression of the *kitla::JRed* fusion protein. DNA (100 pg) was injected directly into wild-type and *kit^b5^* embryos at the one- and two-cell stage for our analysis, using 1% Phenol Red as a tracer. Alternatively, we injected 5′-capped mRNA (30 pg) made from this vector (mMessage mMachine; Ambion, http://www.ambion.com). For simplicity, we refer to embryos injected with either the mRNA or the vector as “*kitla* injected,” unless otherwise noted.

### Melanocyte counts.

Melanocyte migration was determined by counting melanocytes at 48 hpf along seven distinct regions of the embryo: the yolk, near the ear, along the head anterior to the eye, and at each of the four stripes above the hind yolk. Melanocytes on the yolk, head, ventral, and yolk sac stripes were classified as migrated melanocytes and are colored in green in Figure 4H. Those that remained near the ear and on the dorsal and lateral trunk stripes, marked as red in Figure 4H, were considered nonmigratory. Melanocyte survival was determined by counting the total number of melanocytes of larvae between 4 dpf and 11 dpf. Hyperpigmented animals from *kitla* overexpression were placed in 1% epinephrine to retract the melanosomes, and dorsal stripe melanocytes were counted and compared with the number of dorsal melanocytes on a wild-type embryo.

## Supporting Information

Dataset S1 Multiple Sequence Alignment of *kit* Ligands (83 KB RTF)

### Accession Numbers

GenBank (http://www.ncbi.nlm.nih.gov/Genbank) accession numbers for the genes discussed in this paper are *kitla* (AY929068) and *kitlb* (AY929069). Protein sequences with Entrez protein accession numbers from human *KITLG* (NP_000890), mouse *Kitl* (NP_038626), chick *Kitl* (NP_990461), axolotl *Kitl* (AAD17253), *Xenopus Xsl-1* (AAH44108), and *Xenopus Xsl-2* (AAT66411) can be found at http://www.ncbi.nlm.nih.gov/entrez/query.fcgi?CMD=search&DB=protein.

Protein sequences for Fugu rubripes (fugu)*, Gasterosteus aculeatus* (stickleback), and Oryzias latipes (medaka) were found by searching the predicted protein database of each species and can be found on Ensembl (http://www.ensembl.org). Fugu *kitla* (NEWSINFRUP00000171441), stickleback *kitla* (ENSGACP00000012357), medaka *kitla* (ENSORLP00000016705), and medaka *kitlb* (ENSORLP00000016705).

## Abbreviations

dpf: days postfertilization
hpf: hours postfertilization
*kita*: zebrafish *kit a* receptor
*kitb*: zebrafish *kit b* receptor
*Kitl*: mouse *kit ligand*
*kitla*: zebrafish *kit ligand a*
*kitlb*: zebrafish *kit ligand b*
MO: morpholino oligonucleotide
RACE: rapid amplification of cDNA ends
