# Supplementary material for: Gene Duplication of the Zebrafish kit ligand and Partitioning of Melanocyte Development Functions to kit ligand a
Source: PLoS Genet. 2007 Jan 26;3(1):e17. doi: 10.1371/journal.pgen.0030017 (PMC1781495; doi:10.1371/journal.pgen.0030017)
Supplement: Dataset S1 — (83 KB RTF) [file pgen.0030017.sd001.rtf]

Exon          1    2                                   3
KITLG_Hs    1 MKKTQTWILTCIYLQLLLFNPLVKTEGICRNRVTNNVKDVTKL-VANLPKDYMITLKYVP
Kitl_Mm     1 MKKTQTWIITCIYLQLLLFNPLVKTKEICGNPVTDNVKDITKL-VANLPNDYMITLNYVA
Kitl_Gg     1 MKKAQTWIITCFCLQLLLLNPLVKAQSSCGNPVTDDVNDIAKL-VGNLPNDYLITLKYVP
Kitl_Am     1 MKKTKTWIIICIYLQLLLCVT---FGNPCGNPVTDAVNDIEKL-VGNLPSDYSISLEYVP
Xsl-1       1 MKKTKTWIIICIYLQLFLHC----FGNPCGNPITDAVNDIQKL-VGNLPNDYIMKLKYVP
Xsl-2       1 MKKTKTWIIICINLQLFLHC----FGKPCGNPITDAVNDIPKL-VGNIPNDYNMSVRYVP
kitla_Dr    1 MKKSNIWICTCVHLLLYITVA-AYSIEIG-NPITDDIKKISLL-KQNIPKDYKITVRYIP
kitla_Fr    1 ---------ICVHLLLFITLG-VHSTKFDVNPVTDDISRLSVL-KNNIPKDYKIPLDYIP
kitla_Ga    1 -----IWIRVCVHILLFITLG-VHSSKFDVNPVTDDISRLSIL-RQNIPKDYKIPVNYIP
kitla_Ol    1 ------WIRVCVRFLLLITLG-VPSCTFGSFQITDDISKLSVL-KQNIPRDYNISVRYIP
kitlb_Dr    1 MREVKIGESICVLVLLFSGL--VTCSGVFGSPLTDDVATLDTL-SENIPSDYRIPIKFIT
kitlb_Fr    1 MTPITDDVSKLSSLVRRLTSPTCDSFAHRQQIWDANLSQTVLQ-KQNVPSDYEIPVSSIP
kitlb_Ga    1 VHAGSQLILKATCLLLSLCTSLNICFGKFGTPITDDVSKLSLL-KQNMPSDYEIPVSYIP
kitlb_Ol    1 -------ILQATWFLLSLLLSLKLSCGKFGAPITDDVNKLSVL-KQNIPSDYEIPVSYIP

                   4
KITLG_Hs   60 GMDVLPSHCWISEMVVQLSDSLTDLLDKFSNISEG---LSNYSIIDKLVNIVDDLVECVK
Kitl_Mm    60 GMDVLPSHCWLRDMVIQLSLSLTTLLDKFSNISEG---LSNYSIIDKLGKIVDDLVLCME
Kitl_Gg    60 KMDSLPNHCWLHLMVPEFSRSLHNLLQKFSDISDMSDVLSNYSIINNLTRIINDLMACLA
Kitl_Am    57 DMPSLPKQCWVYLMVHKVSNSLESLIHKFANTSQN------YSIMSNLTAILHGIRNCLA
Xsl-1      56 KKESLPKHCWLYMMVVEMTRHLDNLLTKFENTSQN------FLIIKNLSLILQGIRICIR
Xsl-2      56 EKDGLPKHCWLYMMVVEMTRHINELSTKFENTSQN------HLIIYNLSRILQGIRQCFP
kitla_Dr   58 K--EVSGMCWVKLNVFHLEVSLKGLAQKFGNISSN------KDNIGTFVQILQDMRYHIG
kitla_Fr   50 K--ATGGMCWVKLNVFCLENSLHNLSHTFGNISSN------RKDLSIFIKMFQELRFNLE
kitla_Ga   54 R--EEGGMCWVKLNVFYLEESLKGLAHKFGNISSN------RKDISIFIQMFQELRLNMG
kitla_Ol   53 K--ELAGMCWVKLNIFFMEESLNELAKKFGNVSSN------KNDIKIFIDMLQYMRLPLG
kitlb_Dr   58 K--DVGGACWLHLNLYPVESSLKKLAVKFGNQSTN------KANITIFITMLQDFRFTLN
kitlb_Fr   60 K--DVAGTCWVVLNIYPLEQSLRNLAGMFGAVSSN------REQISVFISMLKSLRFTFN
kitlb_Ga   60 K--EVAGPCWVVLNIYPLEQSLRKLASRFGAVSSN------RENTIVFIAMLKSLRFTFD
kitlb_Ol   53 K--EVAGTCWVVLNIYPLEQSLRKLSTMFGAISSN------KDTITVFIAMLKSLRFTFD

                    5                                           ]
KITLG_Hs  117 EN-SSKD-LKKSFKSPEPRLFTPEEFFRIFNRSIDAFKDFVVASETSDCVVSSTLS-PEK
Kitl_Mm   117 EN-APKN-IKESPKRPETRSFTPEEFFSIFNRSIDAFKDFMVASDTSDCVLSSTLG-PEK
Kitl_Gg   120 FD-KNKDFIKENGHLYEEDRFIPENFFRLFNSTIEVYKEFADSLDKNDCIMPSTVETPEN
Kitl_Am   111 SQLIDNEEFITDFPFYDGE-FVPKEYFKYVTKTILLFKAIHKMDDDSTCELPVTTETPLS
Xsl-1     110 ---HDEMDFDSVSSLYQVEGFKPRDFFGYVTSTIEVFKEINNTEYAGPCTMPAEYDFDYD
Xsl-2     110 L--YDEMDFDSASSLYRVEVIKARDFFSYVTSTIEVFKEINNTEYSRTCILPQEYEFEYS
kitla_Dr  110 PG--LEDSMLDFECHYVEEMWLTAKYFEFLEDFFNTANSSRDAED---CEPPPCPTSTKT
kitla_Fr  102 P---VEPIMYEFDCHYRKERWQTAKYFDLVKEFLIAAQNGDNSDD---CEPPPCPTSPRP
kitla_Ga  106 L---LEAIMNDFQCHYREERWQTARYFDFVKDFLIAAQNKEDSDY---CDPPPCPTTPYA
kitla_Ol  105 N---LEPLMYDFECHFRNEQWQTEQYFDYVKDLLKAAEDN-ISDD---CDPPPCPTSLPT
kitlb_Dr  110 SD-DLEDRMQAFKCHYRREKWPTRRFFSYVKSVLTVAGSTYGDIPP--CTPPPCQTLAAP
kitlb_Fr  112 HE-ELEAAMQLFQCHYRERGLMSGLYFDYIKDILHAASQGTSGLP---CKPPPCLNQHPS
kitlb_Ga  112 HE-ELETTMQVFQCHYQEERLMSGLYFDYIKDVLHAASQGASGFS---CKPPSCLNPRQT
kitlb_Ol  105 HE-ELETVMQVFQCHYQEQSLQSSLYFDHIRDVLRAAAQGSSGFS---CKPPPCRNHQQ-

                                6              <c1>               7<c2>
KITLG_Hs  174 -------------------DSR-VSVTKPFMLPPVAASSLRNDSSSSN-------RKAKN
Kitl_Mm   174 -------------------DSR-VSVTKPFMLPPVAASSLRNDSSSSN-------RKAAK
Kitl_Gg   179 -------------------DSR-VAVTKTISFPPVAASSLRNDSIGSNTSSNSN-KEALG
Kitl_Am   170 -------------------DLP-VGVTKPSAKFSFMPSSRKNREGIPN-------AKPDS
Xsl-1     167 -------------------IEA-FQTPSTNHDLPYIPSTRKNSSRFDS-------SARSG
Xsl-2     168 -------------------TEDDFLILDSNHDLPYVPSTRKNSSRFDS-------SARSG
kitla_Dr  165 TITTTTTASTTSAQHSTN---------EKRNGLPDDPEK----------------GAFLL
kitla_Fr  156 VTTEEYLT-------------------ESST---VISSNGPECTTGCTT------YHNPS
kitla_Ga  160 VTTADYLN---------------------------GQIPHHSSSGRVGM------FFFPP
kitla_Ol  158 GNL---------------------------------------------------------
kitlb_Dr  167 PFTP---------------------------------------------------GQSRQ
kitlb_Fr  168 PG-----------------------------------------------------GQEEG
kitlb_Ga  168 PG-----------------------------------------------------GLEEG
kitlb_Ol  160 ----------------------------------DRASS-PDHYHISN-------EKSRG

                                                  8
KITLG_Hs  207 PPGDSS-LHW.AAMALPALFSLIIGFAFGALY.-W-K-KRQP-SLTRA-VENIQIN---E
Kitl_Mm   207 APEDSG-LQWT.AMALPALISLVIGFAFGALY.-W-K-KKQS-SLTRA-VENIQIN---E
Kitl_Gg   218 FISSSS-LQGIS.IALTSLLSLLIGFILGAIY--W.K-KTHPKSRPES-NETIQCH-GCQ
Kitl_Am   203 TSGLA--LETPY.VALISLSSLVLGFIIGVV.C-WKM-KHRE-SGSGC-EPTAPCP-VRK
Xsl-1     200 FNTGAS-IQYST.V-LIALACLVIGFLLGVLFW-W.KFKHRQNQTQDSLSAVAVEP-SAE
Xsl-2     202 FSTGTS-IQYST.V-LIALACLVIGFLLGVL-CLW.KFKHRQTQTQDNLSAVAVEP-RAE
kitla_Dr  200 K------VLESN.LMWLLTIPFAIAVVVLLV--.W-KIKSRRNTPQTDRSPEEGPALFSG
kitla_Fr  188 PLSE---VVERS.LLS-LLFIPL-LALVFLLV--W.KVRSRRNREDMEQDSGERGC-FTG
kitla_Ga  187 EPETLS-GVLEQ.SLLSLLFIPL-VALIFLLV-.W-KVRSCRNEE-NLQQSPGEGGLFPG
kitla_Ol      ------------------------------------------------------------
kitlb_Dr  176 QNGM---NSAVH.GLLALLIIPS-VAILVLTI---.QMALGRRGR-CGARMREIEPHDRA
kitlb_Fr  175 RG-----SSWSIRAP.WILVLIPFTACAVILL--WLG.KSGRLSPSCNVEDLRLRPFDMI
kitlb_Ga  175 RE-----TSWSKRTP.LLLALIPFMACVVVLVYLL--.KSGRLLPVCNTENSHMAPSDTI
kitlb_Ol  178 HS-----WLMRS.PLL-LVLIPF-TTCVVLIV--W.QVR---------------------

                     9
KITLG_Hs  256 EDNEIS-MLQEKEREF-QE-----V----------------
Kitl_Mm   256 EDNEIS-MLQQKEREF-QE-----V----------------
Kitl_Gg   270 EENEIS-MLQQKEKEHLQ------V----------------
Kitl_Am   254 EAEQAS-MLNQTGKAVHL------V----------------
Xsl-1     254 NESQY--ILQQATTDVG-------VI---------------
Xsl-2     256 NESRH--ILQLA---K----IIS-V----------------
kitla_Dr  249 EEANIS-PLDVGISEKNRLNIIMDV----------------
kitla_Fr  238 AEGTAP-PLDADISEK-------------------------
kitla_Ga  240 AEATAP-PLDTEISEKNMLNVIEI-----------------
kitla_Ol      -----------------------------------------
kitlb_Dr  226 EENRNE--LHSGAAQEDPASTSASEQDRAWLDSLGCADTEV
kitlb_Fr  226 PTVSISIPLQTLPNPAD----SEPVQDSVREHESS------
kitlb_Ga  226 ATVSVSIPLQTLTHAAD----TQPVGEAIPEHESG------
kitlb_Ol      -----------------------------------------


Supplemental Figure 1 

Multiple sequence alignment of Kitl sequences from tetrapod and teleost. Black residues indicate that sequence is conserved across at least 40% of taxa, gray boxes indicate similarity across at least 40% of taxa. Each exon is labeled at the first residue of above the human sequence. Only the alignment 5' to exon 6 was used to determine phylogenetic tree (labeled by bracket, ]). Exon 6 appears present in kitla of zebrafish (kitla_Dr), fugu (kitla_Fr), and stickleback (kitla_Ga). Exon 6 also appears present in kitlb of medaka (kitlb_Ol). Medaka kitla is missing exons 6-9, and medaka kitlb is missing 8-9 in the predicted genes. Primary cleavage site, <c1>, and secondary cleavage site, <c2> are not well conserved in the teleost species. 
